# Mac-1 Receptor Clustering Initiates Production of Pro-Inflammatory, Antibacterial Extracellular Vesicles From Neutrophils

**DOI:** 10.3389/fimmu.2021.671995

**Published:** 2021-08-12

**Authors:** Viktória Szeifert, Ferenc Kolonics, Balázs Bartos, Delaram Khamari, Pál Vági, László Barna, Erzsébet Ligeti, Ákos M. Lőrincz

**Affiliations:** ^1^Department of Physiology, Semmelweis University, Budapest, Hungary; ^2^Department of Genetics, Cell- and Immunobiology, Semmelweis University, Budapest, Hungary; ^3^Nikon Center of Excellence, Institute of Experimental Medicine, Hungarian Academy of Sciences, Budapest, Hungary; ^4^Second Department of Internal Medicine, Szent György Hospital, Székesfehérvár, Hungary

**Keywords:** Mac-1, integrins, receptor clustering, neutrophils, extracellular vesicles, pro-inflammatory cytokine, calcium signal

## Abstract

Depending on the prevailing environmental conditions, neutrophilic granulocytes release extracellular vesicles (EV) which have either anti-inflammatory effects on other neutrophils or pro-inflammatory and antibacterial effects. In the present study we investigated the molecular mechanisms underlying the biogenesis of functionally heterogenic EVs. We show that selective stimulation of Mac-1 integrin (complement receptor 3) by specific ligands initiates the generation of EVs which are able to impair bacterial growth and to induce the secretion of the pro-inflammatory cytokine IL-8 (aEV). However, direct Mac-1 stimulation results in aEV release only if neutrophils were activated on ligand coated surfaces whereas soluble ligands are ineffective. Using total internal reflection fluorescence (TIRF) microcopy, an increased clustering of Mac-1 molecules could be visualized in neutrophils added to C3bi coated surfaces; moreover antibody induced cluster formation triggers aEV release as well. Mac-1 induced production of aEV apparently necessitates a strong calcium signal as it fully depends on the presence of extracellular calcium. However, initiation of a strong calcium signal by an ionophore only results the generation of EV devoid of any antibacterial or pro-inflammatory effect. Our results thus demonstrate that stimulation and clustering of Mac-1 is necessary and sufficient for initiation of aEV biogenesis. In contrast, an intracellular calcium signal is necessary but by itself not sufficient for the production of antibacterial and pro-inflammatory EVs.

## Introduction

Extracellular vesicles (EVs) are heterogeneous, phospholipid bilayer-bordered subcellular structures secreted by both pro- and eukaryotic cells spontaneously, upon stimulation or during apoptosis (1). EVs represent a new channel of intercellular communication affecting – among others – also immune cell functions (2–4). EVs are heterogeneous both in size, composition and mechanism of generation (5, 6). Exosomes released from multivesicular bodies are small, have a diameter of (or less than) 100 nm and they are enriched in certain endosome markers such as CD63, CD9 and CD81 tetraspanins (4, 6–8). EVs originating from the cell surface by budding are often referred to as “microvesicles” or “ectosomes” (9, 10). This population is heterogeneous both in size (diameter is typically between 100-1000 nm) and composition that reflects the origin of the EV by containing components of the parent cells (plasma membrane, cytosol and subcellular organelles etc.) (4, 6).

Similar to other cells, neutrophilic granulocyte (polymorphonuclear cell, PMN) produce EVs as well. PMN-derived EVs constitute a regular fraction of EVs in blood (11) that was reported to become significantly elevated in various pathologic conditions (1, 12–14). PMN derived EVs dominantly belong to medium-sized EVs, that shed from the cell membrane (13, 15).

On the basis of our (16–18) and others’ (19, 20) observations we raised the concept that EVs are “tailor-made” whereby their cargo and functional properties are adapted to the prevailing conditions of the environment (21). Resting, non-activated or apoptotic PMNs produce anti-inflammatory EVs and on the other edge if PMNs face the natural enemy the opsonized pathogen, they produce pro-inflammatory EVs that increase the ROS and IL-8 production of resting PMNs and IL-8 secretion of HUVEC cells (16). We and others also demonstrated that EVs issued from opsonized particle activated cells (aEV) had a definitive dose-dependent antibacterial effect (13, 22, 23). We proposed Mac-1 integrin as key factor in switching anti-inflammatory EV generation into production of pro-inflammatory and antibacterial EVs through tyrosine kinase activation and calcium signal (17, 24).

Initiation of a calcium signal by ionophores was also reported to trigger increased EV release from neutrophils (25, 26), however only some specific functional properties of these vesicles were characterized previously.

In the present work our aim was to investigate whether specific activation of the Mac-1 integrin complex or the Ca^2+^-signal on their own are sufficient for the initiation of the aEV biogenesis, and whether these two signals could be additive.

## Methods

### Materials

HBSS buffer with calcium, magnesium and glucose was from GE Healthcare Life Sciences (South Logan, UT, USA), Ficoll-Paque was from GE Healthcare Bio-Sciences AB (Uppsala, Sweden), and dextran was from Serva (Heidelberg, Germany). Zymosan A, bovine serum albumin (BSA), lucigenin (N,N′-dimethyl-9,9′-biacridinium dinitrate), A23187 and DMSO were from Sigma Aldrich (St. Louis, MO, USA). Human complement iC3b and Factor H were from Merck Millipore (Darmstadt, Germany), human fibrinogen were from Calbiochem (San Diego, CA, USA). Triton-X 100 was from Reanal (Budapest, Hungary) and the saponin was from Merck Millipore (Darmstadt, Germany). The conjugated antibodies anti-CD11b-RPE, anti-CD11b-Alexa647 (clone: M1/70) isotype controls and AnnexinV-FITC were from BioLegend (San Diego, CA, USA). The blocking antibodies anti-CD11b (clone: ICRF44) and anti-CD11c (clone: 3.9) were from BioLegend (San Diego, CA, USA). The anti-CD11b-RPE that reacts with an activation-specific epitope of Mac-1 (clone: CBRM1/5) was from ThermoFisher Scientific (Waltham, MA, USA). The non-blocking anti-CD11b (clone: Bear-1) for the artificial cluster induction was from HycultBiotech (Uden, The Netherlands). All other used reagents were of research grade. Green fluorescent protein (GFP) expressing- and chloramphenicol resistant *S. aureus* (USA300) was a kind gift from Professor William Nauseef (University of Iowa, USA).

### Preparation of Human PMN

Venous blood samples were drawn from healthy adult volunteers according to procedures approved by the National Ethical Committee (ETT-TUKEB No. IV/5448-5/EKU). Neutrophils were obtained by dextran sedimentation followed by a 62.5 V/V% Ficoll gradient centrifugation (Beckman Coulter Allegra X-15R, 700 g, 20 min, 22°C). These samples contained more than 95% neutrophils and less than 0.5% eosinophils. Cell viability was above 95% tested by annexin V (less than 5% positivity) and propidium-iodide (less than 1% positivity) labeling. To control the conformation of Mac-1 receptors after the cell preparation we used conformation recognizing antibody (15 μg/ml, clone: CBRM1/5) labeling. Cells were analysed by flow cytometry after 60 min labelling at 4°C. Dominant part of neutrophils carried Mac-1 receptors in high-affinity conformation and their priming could not be further increased by adding TNFα (20 ng/ml, 15 minutes, 37°C). Neutrophils isolated with sterile preparation (pyrogen-free pipette tips and solutions; preparation was carried out in laminar flow hood) had lower percentage of activated Mac-1 receptors and TNFα had a significant effect (**Supplementary Figure 1A**).

### Preparation of PMN Derived EVs

PMNs immediately after preparation (in most cases, 10^7^ cells/mL HBSS) were incubated with or without activating agent for 20 minutes at 37°C in a linear shaking water bath (80 rpm). In experiments where blocking antibodies were used cells were pretreated with the indicated antibodies for 20 minutes at 37°C. Both anti-CD11b (clone: ICRF44) and anti-CD11c (clone: 3.9) were used in 50 µg/mL concentration. After activation, cells were sedimented (500 g, Hermle Z216MK 45° fixed angle rotor, 5 min, 4°C). Upper 500 μL of the supernatant was filtered through a 5 μm pore sterile filter (Sterile Millex Filter Unit, Millipore, Billerica, MA, USA). The filtered fraction was sedimented (15,700 g, Hermle Z216MK 45° fixed angle rotor, 5 min, 4°C) and the pellet was carefully resuspended in the original 500 μL volume. The protein concentration of EV fractions was determined by the Bradford protein assay using BSA as standard in microplate reader (Labsystems iEMS Reader MF; Thermo Scientific, Waltham, MA, USA) at 595 nm.

Spontaneously formed EVs (sEV) were secreted without any activation; stimulation triggered EV were secreted after PMN activation by: 0.5 mg/mL opsonized zymosan A (oZ-EV); 2 µM Ca-ionophore A23187 (Ca-i EV); 20 µg/mL BSA (BSA solub. EV); 50-150 µg/mL C3bi (C3bi solub. EV); 50-150 µg/mL FH (FH solub. EV). In indicated cases, we applied combination of activators. We prepared indicated samples in Ca^2+^-free environment (EV w/o Ca): commercial HBSS without Ca^2+^ and Mg^2+^ was supplemented with 1 mM Mg^2+^. We also activated cells on 6-well plate ligand surface for 20 minutes at 37°C without shaking. Plates were coated by 20 µg/mL BSA (BSA surf. EV); or 50 µg/mL C3bi (C3bi surf. EV); or 50 µg/mL FH (FH surf. EV); or 150 µg/mL fibrinogen overnight at 4°C. In order to avoid the interference of Ca-ionopohore with the bacterial survival measurement, we bound free rest Ca-ionophore by adding BSA in final concentration of 2 mg/mL after the EV initiation period. EVs were analyzed immediately after preparation according to previous data (27).

### Opsonization of Zymosan and Bacteria

Zymosan A (5 mg/mL in HBSS) was opsonized with 500 μL pre-warmed, pooled (derived from 3 donors) human serum for 20 min at 37°C. After opsonization, the zymosan was centrifuged (8,000 g, Hermle Z216MK 45° fixed angle rotor, 5 min, 4°C) and washed in HBSS.

USA300 bacteria (1,000 µl of OD_600nm_=1.0) were opsonized with 200 μL pre-warmed, pooled (derived from 3 donors) human serum for 20 min at 37°C. After opsonization, bacteria were centrifuged (8,000 g, Hermle Z216MK 45° fixed angle rotor, 5 min, 4°C) and washed in HBSS.

### EV Quantification by Flow Cytometry

Human EVs were labeled with RPE conjugated monoclonal anti-CD11b (1 μg/mL), and FITC conjugated annexinV (1 μg/mL) for 20 min at 37°C and then washed in HBSS. The annexinV labeling was controlled in 20 mM EDTA containing medium. For flow cytometric detection of EVs a Becton Dickinson FACSCalibur flow cytometer was used as described previously (17). Briefly, HBSS medium was used for setting the threshold to eliminate instrument noise. Then fluorescent polystyrene beads (3.8 μm SPHERO Rainbow Alignment Particles, Spherotech Inc., USA) were detected to set the upper size limit of EV detection range. EVs were labelled with PE conjugated monoclonal anti-CD11b (1 μg/mL, BioLegend, San Diego, CA, USA, clone M1/70) or FITC conjugated AnnexinV (BD Biosciences). After the measurement of an EV preparation the events of isotype control events (or 20 mM EDTA containing medium in case of annexinV) and the 0.1% TritonX-100 detergent non-sensitive events were subtracted to calculate the true EV number. To avoid swarm detection, the flow rate was held below 1,000 events/s (3,750 events/μL) during measurements. Samples were re-measured after a 2-fold dilution to control linearity of measurements. FC data were analyzed with Flowing 2.5 Software (Turku Center for Biotechnology, Finland).

### Measurement of Size Distribution of EVs by NTA

EV samples were resuspended in 1 ml PBS to reach appropriate particle concentration range for the measurement. Particle size distribution and concentration were analyzed on ZetaView PMX120 instrument (Particle Metrix, Inning am Ammersee, Germany). For each measurement, 11 cell positions were scanned in 2 cycles at 25°C with the following camera settings: shutter speed—100, sensitivity—75, frame rate—7.5, video quality—medium (30 frames). The videos were analyzed by the ZetaView Analyze software 8.05.10 with a minimum area of 5, maximum area of 1,000, and a minimum brightness of 20 (16).

### Bacterial Survival Assay

Opsonized GFP-expressing USA300 bacteria (50 μl of OD_600nm_=1.0) were added to 500 μl of EV (derived from 5*10^6^ PMNs) suspended in HBSS containing 4V/V% LB. After a 40 minutes-long coincubation at 37°C in a linear shaking water bath (80 rpm), we added 2 ml ice-cold stopping solution (0.5 mg/ml saponin in HBSS) in order to stop the incubation and lyse EVs. Then we froze the samples at −80°C for 20 minutes, and thawed to room temperature. The samples were inoculated to LB broth in order to follow the bacterial growth by the measurement of OD in a microplate reader (Labsystems iEMS Reader MF; Thermo Scientific, Waltham, MA, USA) for 8 hours, at 37°C, at 650 nm. At the end, initial bacterial counts were calculated indirectly using an equation similar to PCR calculation, as described previously (28). We also confirmed the results with our previously described flow cytometry based bacterial survival assay (22).

### Measurement of Cytokine Production

PMNs (120 μL of 2.5*10^7^/mL) were added to 480 µl of EV samples at 37°C in a linear shaker (80 rpm) for 3 hours. Cells were centrifuged (500 g, Hermle Z216MK 45° fixed angle rotor, 10 min, 4°C) and supernatants were analyzed for IL-8, TGF-β, IL-1RA, IL-1α, IL-6, TNF-α with a human IL-8, TGF-β, IL-1RA, IL-1α, IL-6, TNF-α DuoSet sandwich ELISA kit according to the manufacturer’s instructions (R&D Systems, Minneapolis, MN, USA) in a microplate reader (Labsystems iEMS Reader MF, Thermo Scientific). We also prepared a control sample for the oZ-EV samples that contained the same amount of zymosan as the oZ-EV isolates. To achieve this, in half of the oZ-EV batch EVs were lysed whereas zymosan particles were left intact (16). We refer to this sample as “lysed oZ-EV”.

### Measurement of Reactive Oxygen Species Production of PMNs

PMNs (200 µl of 1×10^6^/ml) were added onto fibrinogen surface (150 µg/mL) in white flat-bottom 96-well plates. Lucigenin (5 mg/ml dissolved in DMSO) was added in 1:100 volume ratio to the cells. As control of ROS production the inhibitor DPI (5 µM) was used. Changes in the luminescence were recorded for 90 min at 37°C with gentle shaking in a CLARIOstar multi-mode microplate reader (BMG Labtech) every minute.

### TIRF Imaging of Clustering

The #1.5 coverslips (Thermo Scientific, Waltham, MA, USA) were overnight coated with C3bi (50 µg/mL) and BSA (20 µg/mL) at 4°C. The PMNs were first labeled with Alexa647 conjugated monoclonal anti-CD11b (1 μg/mL, clone: M1/70) antibodies for 20 minutes at 37°C and washed in HBSS once. As we demonstrated with ROS measurements, this antibody does not interfere with Mac-1 activation in the used concentration (**Supplementary Figure 1B**). Then 10^5^ cells were placed on BSA or C3bi coated coverslips. Images of the live cells were collected with a Nikon Eclipse Ti2 microscope, using HP Apo TIRF AC 100xH objective lens (numerical aperture=1.49), a HAMAMATSU C13440-20CU ORCA-Flash4.0 V3 camera (2048 (H) × 2048 (V) pixels, 6.5 μm × 6.5 μm pixel size), and a 647 nm, VFL-P-400-647-OEM2-B1 solid state laser. We collected the images in 4 x 4 fields in every 30^th^ seconds with 300 msec exposure time. We made “semi-TIRF images”, as we could not reach flawless TIRF angle during the live experiments. The cells were followed for 20 minutes. We analyzed the images with ImageJ (29) by two different approaches. First, we analyzed the Mac-1 intensities by fluorescence profile measurement along a 25 μm standard line placed on the equator of the cells (**Supplementary Figure 2A**). We ordered in descending sequence the measured intensity values of each cell and we compared the median of these ranked intensities. Second, we carried out a cluster outlining with particle analysis after subtracting the background with 12 μm rolling ball radius and setting the treshold manually to 200 AU minimum intensity (**Supplementary Figures 2B, C**). We measured the maximal fluorescence intensities of all outlined clusters. The median of these peak fluorescence values was used for statistical comparison.

### Induction of Cluster Formation With Antibodies

PMNs were incubated with 10 µg/mL non-inhibitory anti-CD11b (clone: Bear-1) in PBS containing 1% BSA for 30 minutes at 37°C, followed by an incubation with 10 µg/mL mouse anti-human IgG1 for 30 minutes at 37°C. After the clustering induction, the cells were washed once in HBSS and further used for EV generation.

### Statistics

All bar graphs show mean and ± SEM. Difference was taken significant if the P value was <0.05 except the case of multiple one sample t-tests. * represents P < 0.05 and ** represents P < 0.01. In each experiment, “n” indicates the number of independent experiments from different donors. All the analyzed samples showed normal distribution in Shapiro-Wilk normality test. Statistical analysis was performed using GraphPad Prism 8 for Windows (La Jolla, California, USA). We used Sudents t-test to compare pairs of data sets, and ANOVA when we analyzed more than two data sets. As a *post hoc* test for multiple comparisons we used Tukey’s method when every sample was compared with every other sample and Dunnett’s method when every sample was compared with a control sample. We used Sidak’s test to compare some indicated sample pairs.

## Results

### Selective Activation of Mac-1 Induces EV Production in Adherent but Not in Suspended PMNs

**Figure 1:**
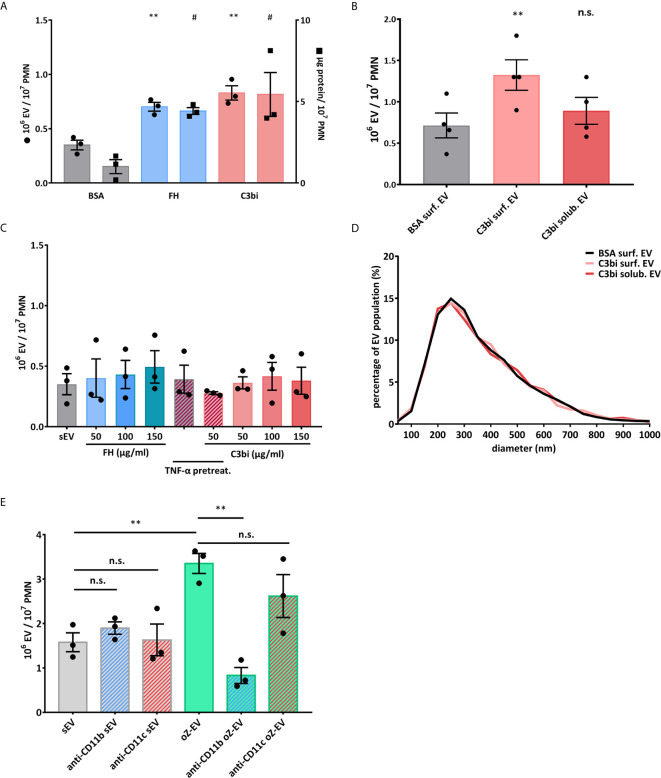
Mac-1 ligand surface induces EV production from adherent PMNs. **(A)** Comparison of EV production of adherent PMNs on BSA and surface-bound Mac-1 ligands. Dot scatter bars represent the EV quantification by flow cytometry, square scatter bars represent the quantification based on protein amount measurement. The EV production was measured on BSA surface (20 µg/ml), on C3bi surface (50 µg/ml) and on FH surface (50 µg/ml) for 20 minutes. N=3, error bars represent mean ± S.E.M. Data were compared to BSA by using RM one-way ANOVA coupled with Dunett’s *post hoc* test. **(B)** Comparison of the concentration of EV samples generated by adherent and soluble PMNs measured by NTA. C3bi (50 µg/ml) and BSA (20 µg/ml) were used in the previously detailed concentration. N=3, error bars represent mean ± S.E.M. Data were compared to BSA surface by using RM one-way ANOVA coupled with Dunett’s multiple comparisons test. **(C)** Comparison of EV production of PMNs induced by soluble Mac-1 ligands in different concentrations. The quantification of the EVs was carried out by flow cytometry. N=3, error bars represent mean ± S.E.M. Data were compared to spontaneous EV production (sEV) by using RM one-way ANOVA coupled with Dunett’s *post hoc* test. **(D)** Representative diagram of size distribution of PMN EVs induced by soluble or surface-bound C3bi measured by NTA. **(E)** Spontaneous and opsonized zymosan induced EV production of suspended PMNs in the presence of anti-CD11b (clone: ICRF44) and anti-CD11c (clone: 3.9) inhibitory antibodies. The quantification of the EVs was carried out by flow cytometry, EVs were labelled with FITC-Annexin V. N=3, error bars represent mean ± S.E.M. Data were compared by using RM one-way ANOVA coupled with Sidak’s multiple comparison test. ^#^P < 0.05 and **P < 0.01; n.s., non significant.

We examined selective activation of Mac-1 by application of its ligands: complement fragment C3bi and factor H (FH). Both flow cytometry based quantification of the EVs (**Figure 1A**) and NTA based concentration measurement (**Figure 1B**) showed that PMNs placed on Mac-1 ligand surfaces increased their EV production significantly compared to EV production on albumin surface. We made similar observations on another Mac-1 ligand fibrinogen surfaces as well (**Supplementary Figure 3**). However neither soluble C3bi, nor soluble FH were able to increase the EV production regardless of the concentration of the ligand or the presence of TNFα that initiates the inside-out activation of Mac-1 (**Figures 1B, C**). There was no difference in the size distribution of the differently initiated EVs (**Figure 1D**). The diameter of the EVs spreads mostly between 100 and 700 nm with a modus around 200-300 nm. As a control we also tested the effect of the complement receptor blocking antibodies on EV production of suspended neutrophils after opsonized zymosan activation (**Figure 1E**). Spontaneous EV production was not affected by using either CR3 (Mac-1) or CR4 blocking antibodies but opsonized zymosan induced EV production was completely inhibited in the presence of CR3 inhibitor. Anti-CR4 antibodies did not interfere significantly with opsonin receptor induced EV production that agrees well with previous observations (17, 30). The dominant role of Mac-1 over CR4 is also supported by ROS measurements in the presence of the different blocking antibodies (**Supplementary Figure 4**).

**Figure 2:**
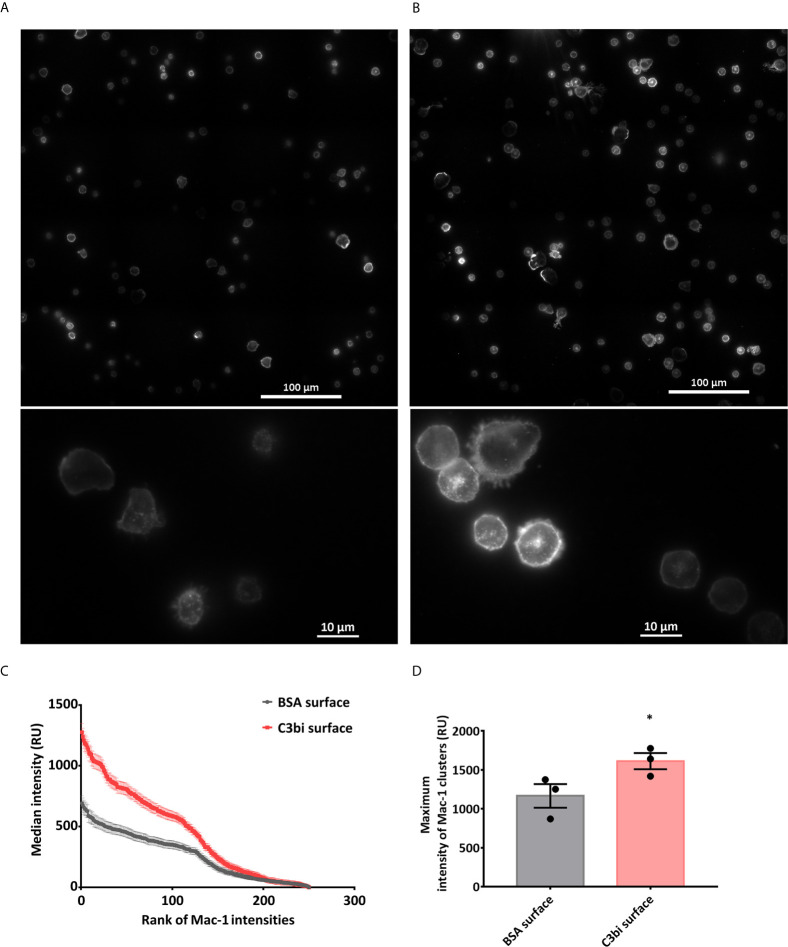
Mac-1 receptor clustering on BSA and C3bi surface. **(A, B)** Representative TIRF microscopic images of the cells after 20 minutes on coated surfaces. Cells were labelled with Alexa647-conjugated anti-CD11b. **(A)** BSA-coated surface, **(B)** C3bi-coated surface captured at the 20^th^ minute time point. **(C)** Median fluorescence intensity values of the ranked intensities of all the cells measured along a standard 25 μm line. Three independent experiments. Error bars represent mean ± S.E.M. Data were compared by linear regression. The two datasets are significantly different (p<0.0001). **(D)** Median of peak intensity values of the clusters in the samples. Three independent experiments, error bars represent mean ± S.E.M. Data were compared by using Students’s t-test. *P < 0.05.

The surprising difference in the effect of Mac-1 activation on a surface or in suspension raises the possibility that Mac-1 does not bind the applied ligands in suspension or that the mere binding of a ligand is not able to trigger EV production. Binding of conformation-specific antibodies indicated that our PMN isolated under non-sterile conditions present Mac-1 mostly in activated state which cannot be significantly increased by TNFα treatment (**Supplementary Figure 1A**). Thus C3bi and FH could bind to Mac-1 of the suspended PMN. On the other hand, it should be recalled that the serum opsonized particles, used as trigger in our earlier experiments (13, 17) also provide a “surface” for the neutrophils. We investigated therefore the possible importance of the Mac-1 receptor clustering in the EV production of the neutrophils. In order to visualize the pattern of Mac-1 receptors during surface activation, we carried out total internal reflection fluorescence (TIRF) microcopy with anti-CD11b (clone M1/70) antibody labelling (**Figures 2A, B** and **Supplementary Videos 1**, **2**). We measured the Mac-1 fluorescence intensity along the equator of each of the cells on C3bi or BSA surface and ranked the values in a descending order. The intensity profiles of the median values of the ranks of the samples showed significantly different curves. The intensity profile on C3bi surface demonstrates higher Mac-1 receptor density on these cells than on BSA adhered cells (**Figure 2C**). We also examined the peak Mac-1 receptor density of the Mac-1 clusters of cells adherent to the two different surfaces. The median values of the peak intensities were higher in case of C3bi surface (**Figure 2D**).

### Dependence of the Functions of the PMN EVs on Mac-1 Clustering

**Figure 3:**
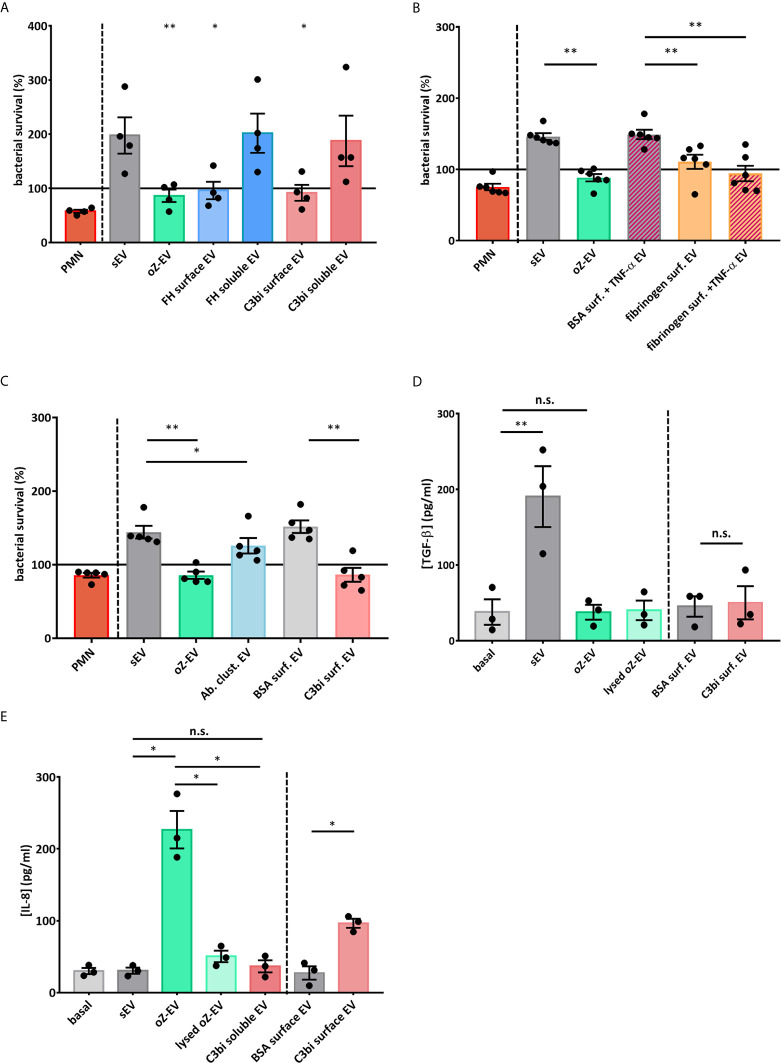
Mac-1 ligand surface induce antibacterial and pro-inflammatory PMN EV production **(A)** Bacterial survival in the presence of different types of neutrophil EVs. The amount of the applied EVs was normalized to protein content. The bacterial survival of *S. aureus* was quantified by optical density-based method. N=4, error bars represent mean ± S.E.M. Data were compared to sEV by using RM one-way ANOVA coupled with Dunett’s *post hoc* test. **(B)** Bacterial survival in the presence of fibrinogen surface induced neutrophil EVs. The amount of the applied EVs was normalized to protein content. N=6, error bars represent mean ± S.E.M. Data were compared to sEV by using RM one-way ANOVA coupled with Sidak’s multiple comparison test. **(C)** Bacterial survival in the presence of EVs produced after antibody triggered cluster formation (Ab clust. EV). The amount of the applied EVs was normalized to protein content. N=5, error bars represent mean ± S.E.M. Data were compared to sEV by using RM one-way ANOVA coupled with Sidak’s multiple comparison test. **(D)** PMNs were treated for 3 h with different PMN EV populations or with controls. TGF-β amount of the supernatant was quantified with ELISA. N=3, error bars represent mean ± S.E.M. Data were compared by using RM one-way ANOVA coupled with Sidak’s multiple comparison test. **(E)** PMNs were treated for 3 h with different PMN EV populations or with controls. IL-8 amount of the supernatant was quantified with ELISA. N=3, error bars represent mean ± S.E.M. Data were compared by using RM one-way ANOVA coupled with Sidak’s multiple comparison test. *P < 0.05 and **P < 0.01. n.s., non-significant.

Next we examined the functionality of the C3bi induced EVs. We analyzed the bacterial survival of *S. aureu*s strains after 40 minutes co-incubation with the different EV populations. We found that only the EVs produced on Mac-1 ligand surfaces showed similar bacterial growth inhibiting effect as the previously observed serum opsonized zymosan induced EVs (oZ-EVs) (**Figures 3A, B**). We did not observe any antibacterial effect of spontaneously formed EV and soluble Mac-1 ligand induced EVs.

To test whether forced clustering of Mac-1 receptors on the surface of suspended PMN is able to induce antibacterial EV production, we carried out antibody induced clustering with a non-inhibitory Mac-1 antibody clone. The EVs isolated 30 minutes after the addition of secondary antibodies showed significant antibacterial effect (**Figure 3C**). The measured effect was weaker than the effect of oZ-EV or C3bi surface triggered EVs.

We also measured the production of the pro-inflammatory cytokines IL-1α, IL-8, IL-6, TNF-α and anti-inflammatory cytokines TGF-β and IL-1RA by PMNs during 3 hours co-incubation with the differently triggered EV populations. Spontaneously produced EVs triggered TGF-β (**Figure 3D**), whereas opsonized zymosan induced EVs elicited a clear IL-8 answer from PMNs. As control we applied lysed oZ-EV samples to test the possible effect of remnant zymosan particles. Importantly, the EVs produced on C3bi-coated surface resulted significantly higher IL-8 production than EVs that were harvested on BSA surface. In contrast soluble C3bi triggered EVs did not increase the IL-8 production of resting PMNs (**Figure 3E**). In case of IL-1α, IL-6, TNF-α and IL-1RA PMN did not secrete detectable quantity (**Supplementary Figure 5**).

### Role of Extracellular Ca^2+^ in the Generation of PMN EVs 

**Figure 4:**
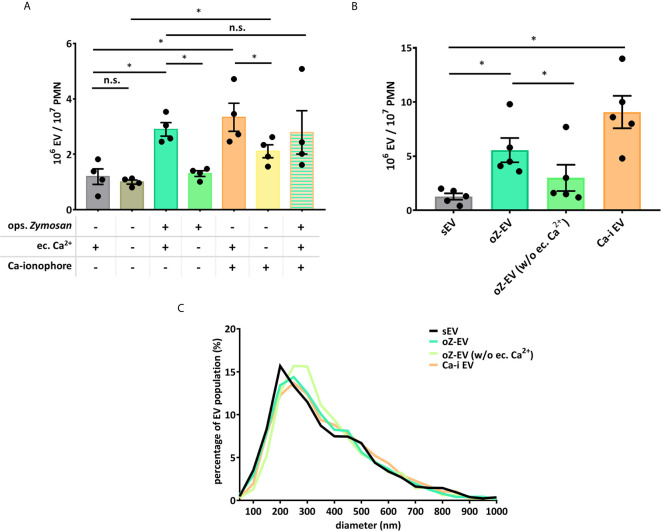
Role of the Ca^2+^ supply in the PMNs’ EV production. **(A)** Comparison of EV production of PMNs quantified by flow cytometry. Dependence on Ca^2+^ supply was tested by omitting Ca^2+^ from the incubation medium or by addition of A23187 Ca-ionophore. N=4, error bars represent mean ± S.E.M. Data were compared by using RM one-way ANOVA coupled with Sidak’s *post hoc* test. **(B)** Comparison of the concentration of the differently induced PMN EVs, measured by NTA. N=3, error bars represent mean ± S.E.M. Data were compared by using RM one-way ANOVA coupled with Sidak’s multiple comparisons test. “Ca-I EV” signifies EVs produced in the presence of Ca-ionophore and extracellular calcium. **(C)** Representative diagram of size distribution of PMN EVs produced in different conditions measured by NTA. *P < 0.05; n.s., non significant.

Our earlier work showed that Ca^2+^ supply is important for the generation of PMN EVs (24). In order to answer the question, whether the Ca^2+^ signal itself is sufficient for the aEV generation, we tested the effect of the Ca-ionophore A23187, and the absence of extracellular Ca^2+^ (**Figures 4A, B**). Based on the quantification of the EVs, we found that the absence of extracellular Ca^2+^ does not affect the spontaneous EV release of the cells, but the oZ-EV production did decrease significantly. Ca-ionophore triggered a strong EV generation in the presence of extracellular Ca^2+^ that was partially inhibited by the withdrawal of extracellular Ca^2+^. We also tested the combination of the opsonized zymosan and Ca-ionophore application on neutrophils, but the Ca-ionophore could not further potentiate the oZ-EV generation (**Figures 4A, B**). Data obtained with FC (**Figure 4A**) are in good agreement with the results obtained with NTA (**Figure 4B**). The size distribution of the EVs was not changed by the presence of the Ca-ionophore or the absence of extracellular Ca^2+^ supply (**Figure 4C**), the size of the EVs varies in the 100-700 nm range, with a peak around 200-300 nm.

### Effect of the Presence of Extracellular Ca^2+^ on the Functions of the PMN EVs

**Figure 5:**
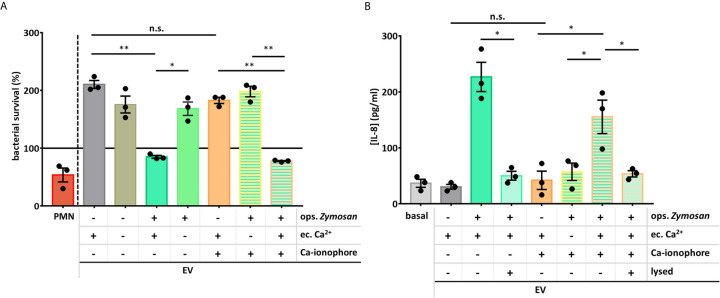
Extracellular Ca^2+^ is necessary for the PMNs’ antibacterial and pro-inflammatory EV production. **(A)** Bacterial survival in the presence of different types of neutrophil EVs. The amount of the applied EVs was normalized to protein content. The bacterial survival of *S. aureus* was quantified by optical density-based method. N=3, error bars represent mean ± S.E.M. Data were compared by using RM one-way ANOVA coupled with Sidak’s *post hoc* test. **(B)** PMNs were treated for 3 h with different PMN EV populations or with controls. IL-8 amount of the supernatant was quantified with ELISA. N=3, error bars represent mean ± S.E.M. Data were compared by using RM one-way ANOVA coupled with Sidak’s multiple comparison test. *P < 0.05 and **P < 0.01. n.s., non-significant.

We examined the functionality of the PMN EVs produced in the absence of extracellular Ca^2+^ or in the presence of Ca-ionophore. We found that only those EVs could decrease the survival of *S. aureus* that were produced in the presence of extracellular Ca^2+^ and were activated by opsonized zymosan (**Figure 5A**), all other EV types showed no effect, similar to the spontaneously generated EVs.

Similar pattern was seen in the IL-8 production. Both the presence of extracellular Ca^2+^ and the Mac-1 stimulator zymosan were needed to generate pro-inflammatory EVs. These data also support that the application of Ca-ionophore with or without extracellular Ca^2+^ cannot result EVs with the same effect as the oZ-EVs and cannot potentiate the effect of the oZ-EVs either.

## Discussion

In earlier experiments, using opsonized bacteria or zymosan particles as a trigger, we demonstrated the key role of the β2 integrin Mac-1 (that also functions as complement receptor CR3) in generation of EVs with antibacterial properties (aEV) (17). Later we also showed that aEVs have pro-inflammatory effect on other neutrophils (16). In those experiments an auxiliary role of other receptors (e.g. pattern recognition receptors) could not be excluded. In the present study we applied only selective ligands and inhibitory antibodies of Mac-1 and demonstrate that specific stimulation of Mac-1 alone is sufficient for changing the generation of anti-inflammatory sEVs (**Figure 3D**) into antibacterial (**Figures 3A–C**) and pro-inflammatory (**Figure 3E**) aEV. The investigated ligands (C3bi, factor H and fibrinogen) were similarly effective (**Figures 1A, B** and **3A, B**). However, Mac-1 stimulation only initiated aEV generation if PMN were added to a surface coated with the specific ligands (**Figures 1A, C**), whereas no increase in EV release was observed if either ligand has been applied in soluble form even in very high concentration (**Figures 1B, C**). Identical data were obtained independent of the method of detection (FC or NTA).

The observed difference in the effectivity of Mac-1 ligands (whether applied in soluble form or on a solid surface) raised the possibility that clustering of Mac-1 integrins is necessary for aEV formation. Indeed, TIRF microscopy revealed higher average intensity of fluorescently labelled Mac-1 molecules in PMN seated on a C3bi than on BSA surface (**Figures 2A–C**). In addition to the average intensity also the maximal intensity of fluorescently labelled clusters was elevated in PMNs on C3bi as compared to BSA surface (**Figure 2D**). The fluorescence microscopic images indicate a strong concentration of Mac-1 molecules in the cell membrane representing increased clustering of Mac-1 molecules on the C3bi surface. The data of antibody induced clustering proves that clustered Mac-1 molecules are able to attract the signaling elements required for initiation of the generation of aEVs with antibacterial properties. Clustering of the receptors was showed to be critical for signal transduction in case of several other immune receptors, such as BCR (31), TCR (32) and Mac-1 initiated phagocytosis (33–35).

Production of aEVs was critically dependent on the presence of Ca^2+^ in the extracellular space. In the absence of added calcium, both the number of released EVs was significantly lower than in its presence (**Figures 4A, B**) and the antibacterial and IL-8 production enhancing effects were absent (**Figure 5A**). Apparently a strong Ca^2+^ signal involving Ca^2+^ entry from the extracellular space is required to direct aEV formation. However, our results obtained with the Ca^2+^ ionophore indicate that the strong Ca^2+^ signal on its own does not lead to aEV formation. In the presence of external Ca^2+^ the ionophore was able to initiate increased EV formation, the amount of which was comparable to the effect of Mac-1 stimulation, or even exceeded it (**Figures 4A, B**). However, these EVs were devoid of antibacterial effect and did not initiate cytokine release (**Figures 5A, B**). Interestingly, the Ca^2+^ ionophore was able to initiate significant EV production also in the absence of external Ca^2+^, probably by mobilization of internal sources. This observation suggests that the machinery itself that leads to physical formation of EVs depends strongly on Ca^2+^ signaling independent from the source of Ca^2+^ (36–38). However, the equipment of EVs with antibacterial and pro-inflammatory properties depends on signaling processes of different Ca^2+^ sensitivity or needs long-lasting Ca^2+^ signal or store operated refill of intracellular Ca^2+^ stores. Finally, combined application of Mac-1 stimulation with the A23187 indicates that the ionophore is not able to further augment the effect of the Mac-1 stimulation itself.

It should be noted that absence of extracellular Ca^2+^ had no effect either on the number (**Figure 4A**) or on the biological activity (**Figure 5A**) of EVs released from resting PMN spontaneously (sEV). In previous studies we observed that inhibition or genetic deletion of various tyrosine kinases or phospholipase C had no effect either (17). Release of sEV with anti-inflammatory properties seems to be an inherent constitutive activity of PMNs and probably also other cells.

Taken together, our study proves that stimulation of Mac-1 by itself is sufficient to initiate production of antibacterial and pro-inflammatory EVs, provided clusterization can occur. In contrast, calcium entry is necessary, but on its own not sufficient for aEV biogenesis. We thus provide the first time a definitive link between specific molecular trigger acting on an identified receptor and the production of EVs with distinctive functional properties. These new results further support our earlier (16, 21) suggestion that EVs are “tailor-made” depending on the environmental conditions prevailing at the time of their generation.

## Data Availability Statement

The raw data supporting the conclusions of this article will be made available by the authors, without undue reservation.

## Ethics Statement

The studies involving human participants were reviewed and approved by ETT-TUKEB No. IV/5448-5/EKU. The patients/participants provided their written informed consent to participate in this study.

## Author Contributions

VS carried out most experiments, prepared the figures and wrote part of the manuscript. FK worked out the methodology of cytokine measurements. BB worked out the methodology and carried out some of fibrinogen surface measurements. DK helped with the NTA measurements, PV and LB helped with the microscopy. ÁL and EL designed and supervised the study and wrote the manuscript, EL obtained funding. All authors contributed to the article and approved the submitted version.

## Funding

Experimental work was supported by research grant No. 119236 from NKFIH and 2.3.2.-16 from VEKOP to EL. This paper was supported by the János Bolyai Research Scholarship of the Hungarian Academy of Sciences to ÁL, and by the ÚNKP-20-5 New National Excellence Program of the Ministry for Innovation and Technology from the source of the National Research, Development and Innovation Fund to ÁL.

## Conflict of Interest

The authors declare that the research was conducted in the absence of any commercial or financial relationships that could be construed as a potential conflict of interest.

## Publisher’s Note

All claims expressed in this article are solely those of the authors and do not necessarily represent those of their affiliated organizations, or those of the publisher, the editors and the reviewers. Any product that may be evaluated in this article, or claim that may be made by its manufacturer, is not guaranteed or endorsed by the publisher.

## Supplementary Material

Supplementary Figure 1 **(A)** Presentation of the conformation of Mac-1 integrins on neutrophils with or without TNF-α pre-treatment with conformation recognizing antibody (clone: CBRM1/5) labeling. Non-sterile cell preparation (marked as ‘PMN’) that was used in this study primes neutrophils and brings Mac-1 receptors into high-affinity conformation. Dot scatter bars represent the percent of cells that were positive for CBRM1/5 labeling. N=3, error bars represent mean ± S.E.M. Data were compared by using RM one-way ANOVA coupled with Sidak’s multiple comparison test. **(B)** Comparison of ROS production in the presence of increasing concentration of anti-CD11b (M1/70 clone) and inhibitory anti-CD11b (clone: ICRF44) antibodies. Peak ROS production is presented. N=3, error bars represent mean ± S.E.M. Data were compared to maximal ROS production of PMNs on fibrinogen surface. Data were analysed by multiple one sample t-tests.

Supplementary Figure 2 **(A)** Intensity profile measurement of the cells. We measured the fluorescence profile along a 25 μm standard line placed on the equator of the cells. **(B, C)** Cluster intensity measurement of the cells. We carried out particle analysis after subtracting the background with 12 μm rolling ball radius and setting the threshold manually to 200 AU minimum intensity in order to outline the clusters. We measured the maximal fluorescent intensities of all outlined clusters on the native image.

Supplementary Figure 3 Comparison of EV production of adherent PMNs on BSA or fibrinogen surface quantificated by flow cytometry. The EV production was measured on BSA surface (20 µg/ml) and on fibrinogen surface (150 µg/ml) for 20 minutes. N=13, error bars represent mean ± S.E.M. Data were compared by using paired Students’s t-test.

Supplementary Figure 4 Comparison of ROS production of PMNs placed on fibrinogen surface in the presence of anti-CD11b (clone: ICRF44) and anti-CD11c (clone: 3.9) inhibitory antibodies. Peak ROS production is presented. N=4, error bars represent mean ± S.E.M. Data were compared to maximal ROS production of PMNs on fibrinogen surface. Data were analysed by multiple one sample t-tests.

Supplementary Figure 5 Cytokine production of EV stimulated PMNs and monocytes. Cells were treated for 3 h with different PMN EV populations or with controls. The cytokine amount of the supernatant was quantified with ELISA. N=3, error bars represent mean ± S.E.M. Data were compared by using RM one-way ANOVA coupled with Tukey’s multiple comparison test. To demonstrate that the assay is working, we measured monocyte control samples as well, N=1.

Supplementary Video 1 Total internal reflection fluorescence (TIRF) video of anti-CD11b labelled PMNs placed on BSA surface. Cells were followed for 20 minutes, data were collected in every 30 seconds. Scale bar: 20 μm.

Supplementary Video 2 Total internal reflection fluorescence (TIRF) video of anti-CD11b labelled PMNs placed on C3bi surface. Cells were followed for 20 minutes, data were collected in every 30 seconds. Scale bar: 20 μm
