# Supplementary figures and images for: Mac-1 Receptor Clustering Initiates Production of Pro-Inflammatory, Antibacterial Extracellular Vesicles From Neutrophils
Source: Front Immunol. 2021 Aug 12;12:671995. doi: 10.3389/fimmu.2021.671995 (PMC8397541; doi:10.3389/fimmu.2021.671995)

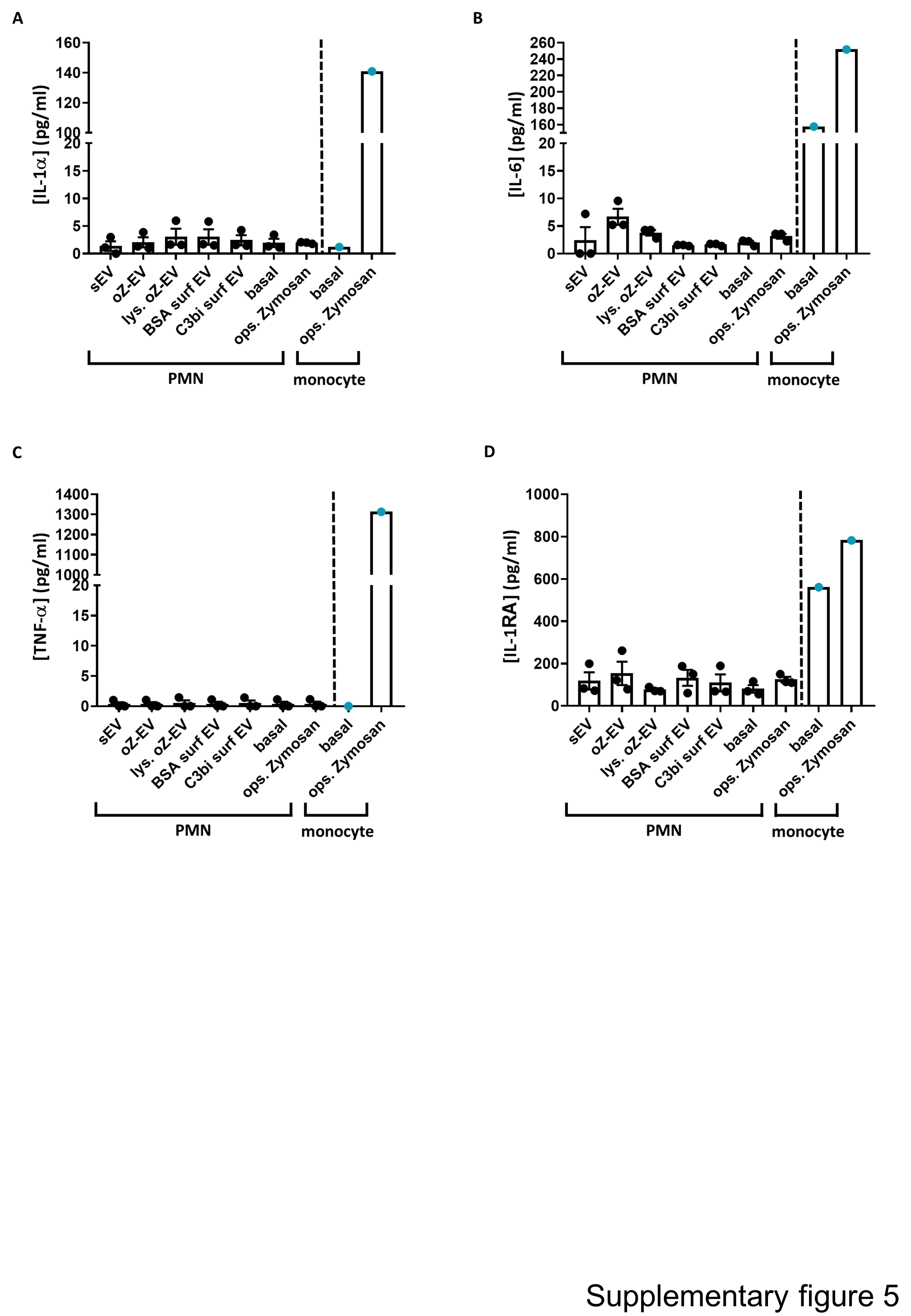

Supplement: Supplementary Figure 5 — Cytokine production of EV stimulated PMNs and monocytes. Cells were treated for 3 h with different PMN EV populations or with controls. The cytokine amount of the supernatant was quantified with ELISA. N=3, error bars represent mean ± S.E.M. Data were compared by using RM one-way ANOVA coupled with Tukey’s multiple comparison test. To demonstrate that the assay is working, we measured monocyte control samples as well, N=1. [file Image_5.tif]
